# Dental Aspect of Distal Tubular Renal Acidosis with Genu Valgum Secondary to Rickets: A Case Report

**DOI:** 10.1155/2012/374945

**Published:** 2012-04-01

**Authors:** Rakesh N. Bahadure, Nilima Thosar, Ritika Kriplani, Sudhindra Baliga, Punit Fulzele

**Affiliations:** Department of Pedodontics and Preventive Dentistry, Sharad Pawar Dental College, Sawangi (M), Mahartashtra State, Wardha 442102, India

## Abstract

Distal renal tubular acidosis is a disease that occurs when the kidneys do not remove acid properly into the urine, leaving the blood too acidic (called acidosis). Distal renal tubular acidosis (type I RTA) is caused by a defect in the kidney tubes that causes acid to build up in the bloodstream. It ultimately results rickets which include chronic skeletal pain, in skeletal deformities, skeletal fractures. Rickets is among the most frequent childhood diseases in many developing countries. Dental problems in rickets include delayed eruption of permanent teeth, premature fall of deciduous teeth, defects in structure of teeth, enamel defects in permanent teeth (hypoplastic), pulp defects, intraglobular dentine, and caries tooth. Herewith, reported a case of distal tubular renal acidosis with genu valgum secondary to rickets, with pain and extraoral swelling associated with right and left mandibular 1st permanent molars. Teeth were infected with pulp without being involved with caries. Radiographically cracks in enamel and dentin were observed. Pulp revascularization with 46 and root canal treatment was done for 36 with followup of 1 year.

## 1. Introduction

Rickets is a softening of bones in children due to deficiency or impaired metabolism of vitamin D, magnesium, phosphorus or calcium [1], potentially leading to fractures and deformity. The origin of the word “rickets” is probably from the Old English dialect word “wrickken,” to twist. The Greek derived word “rachitis” (meaning “inflammation of the spine”) was later adopted as the scientific term for rickets, due chiefly to the words' similarity in sound. Alternate names for rickets include Osteomalacia in children; vitamin D deficiency; Renal rickets; Hepatic rickets.

Although described earlier, distal renal tubular acidosis was recognized as a distinct entity by Albright et al. [2] in 1946. The clinical features described consisted of hypokalemia, hyperchloremic metabolic acidosis, inability to lower urine pH below 5.5, nephrocalcinosis, and nephrolithiasis. The syndrome was designated “distal renal tubular acidosis,” since the establishment of a large pH gradient between urine and blood is a function of the distal nephron. Primary distal renal tubular acidosis (DRTA) is characterized by metabolic acidosis of varying severity accompanied by inappropriately alkaline urine. Other features include low serum potassium due to renal potassium wasting and elevated urinary calcium. If untreated, this acidosis may result in dissolution of bone, leading to osteomalacia and rickets [3].

Genu valgum is the Latin-derived term used to describe knock-knee deformity. These symptoms reflect the pathologic strain on the knee and its patellofemoral extensor mechanism. In this paper, dental management is described who was diagnosed as a case of distal tubular renal acidosis with genu valgum secondary to rickets.

## 2. Case Report

**Figure 1:**
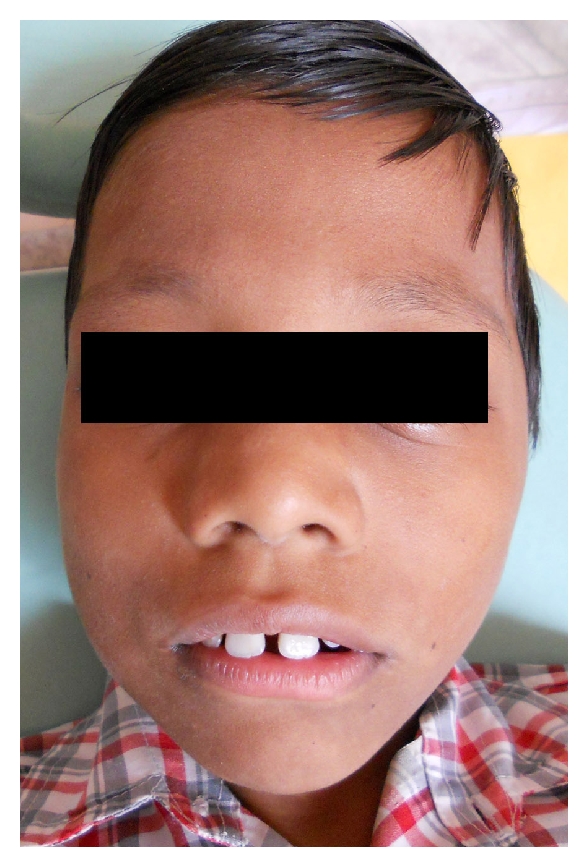
Showing extra oral swelling on right side of face.

**Figure 2:**
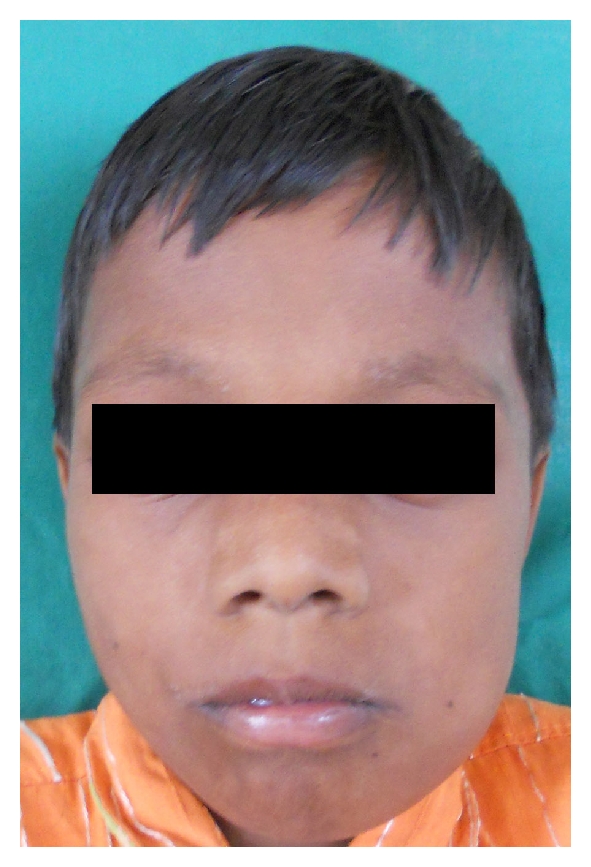
Showing extra oral swelling on left side of face.

**Figure 3:**
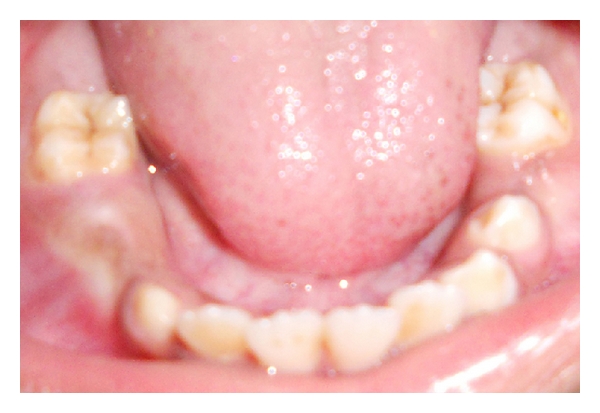
Showing sound tooth structure of 36 and 46 clinically without being involved with caries.

**Figure 4:**
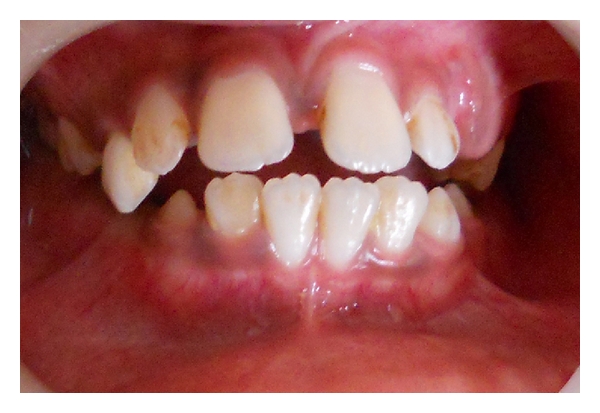
Showing open bite.

A 9-year-old male patient reported to the Department of Pedodontics and Preventive Dentistry with chief complaint of pain and extraoral swelling in lower right posterior region. Family history was noncontributory. 4 days later patient again reported with extraoral swelling in lower left posterior region (Figures 1 and 2). On intraoral examination, mandibular right and left first permanent molars were sound without being involved with caries (Figure 3). Teeth present were 11, 12, 55, 16, 21, 22, 23, 65, 26, 31, 32, 33, 36, 41, 42, 43, 46. Early exfoliation of deciduous teeth was seen. Patient also showed open bite (Figure 4). 

**Figure 5:**
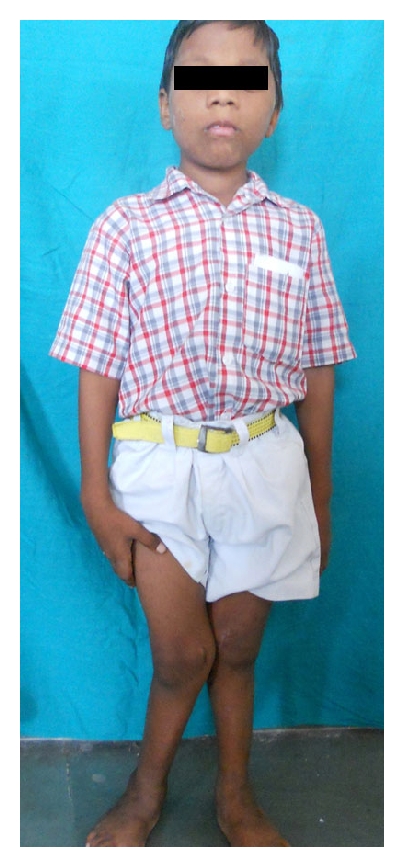
Showing genu valgum.

Physical examination showed the patient as being with a short stature and waddling gait, weight of 14 kg and a height of 2 feet 6 inch. Lower limbs showed Genu Valgum (knock-knee) deformity (Figure 5). Scar was seen on bilateral knees. Medical records suggested that there was 30° genu valgum in right knee and 25° in left knee. Bilateral medial maleollus distance was 20 cm. Bilateral procurvature deformity was around 15°, and deformity disappears on flexing knees. History related to development suggests that patient could stand at the age of 2 yrs and walk at the age of 3.5 yrs. There was no similar history in any other sibling in the family.

Medical history revealed that the patient was a case of distal tubular renal acidosis with bilateral genu valgum secondary to rickets. Patient's medical record showed that there was history of pain in both limbs since 1.5 yrs and inward bowing of knees since 1.5 yrs. Patient was admitted 1 year back and was on oral calcium tablets.

Laboratory investigation revealed the following: urine calcium 12.6 mg%, urine creatinine 55.2 mg%, serum calcium 12.2 mg%, serum chloride 62.6 mEq/L, inorganic phosphate 4.2 mg%, serum potassium 17.7 mEq/L (normal range in male: 3–5 mEq/L), serum sodium 83 mEq/L (normal range in male: 136–145 mEq/L), and serum phosphorous 3.1 mg%. Alkaline phosphatase level was 1544 IU/L.

**Figure 6:**
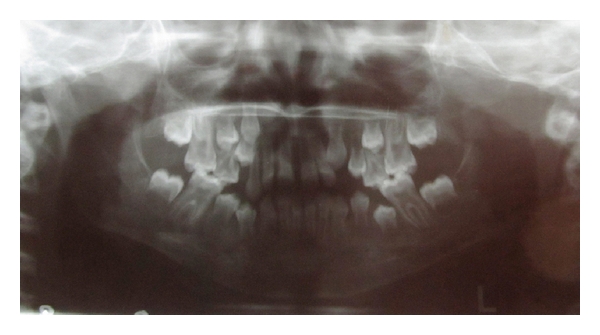
Showing orthopantomogram with apical involvement of 36 and 46.

Radiographic examination showed radiolucencies in enamel and dentin and cracks in enamel and dentin extending to the pulp. Also periapical lesion was present associated with 36, 46. Root apices of 36, 46 were not complete (Figure 6).

Pulp vitality test showed 36 and 46 were nonvital.

Depending upon signs and symptoms, pulp revascularization for 46 and root canal treatment for 36 was considered. For pulp revascularization of 46, triple antibiotic paste was used consisting of ciprofloxacin, metronidazole, and tetracycline mixed with normal saline. The paste was kept in the canal for 11 days. It was later on replaced by mineral trioxide aggregate. Root canal treatment was carried for 36. Remaining teeth were treated with pit and fissure sealants and topical fluoride as a preventive measure.

## 3. Discussion

Mineralization of bone matrix depends on the presence of adequate supplies of 1, 25-dihydroxy vitamin D (1, 25(OH)_2_D), calcium, phosphorus, and alkaline phosphatase, and on a normal body pH. If there is a deficiency of any of these substances, or if there is severe systemic acidosis, the mineralization of bone will be defective. This results in a qualitative abnormality of bone, with a reduction in the mineral to osteoid ratio, resulting in rickets in children and osteomalacia in adults [4]. Chronic acidosis can lead to decreased bone mineral content [5, 6].

Rickets is a childhood disease characterized by impeded growth and deformity of the long bones.

Renal tubular acidosis may also interfere with the process of mineralization and cause rickets. Rickets can only occur in the presence of unfused epiphyses as it manifests itself in the growth plate [7].

Vitamin D helps the body control calcium and phosphate levels. If the blood levels of these minerals become too low, the body may produce hormones that cause calcium and phosphate to be released from the bones. This leads to weak and soft bones.

Hereditary rickets is a form of the disease that is passed down through families. It occurs when the kidneys are unable to hold onto the mineral phosphate. Rickets may also be caused by kidney disorders that involve renal tubular acidosis.

Disorders that reduce the digestion or absorption of fats will make it more difficult for vitamin D to be absorbed into the body. Occasionally, rickets may occur in children who have disorders of the liver or who cannot convert vitamin D to its active form. Role of liver causing rickets was not applicable to the present case.

Symptoms associated with rickets include bone pain or tenderness in arms, legs, pelvis, spine, dental deformities like delayed formation of teeth, decreased muscle tone (loss of muscle strength), defects in the structure of teeth; holes in the enamel, increased cavities in the teeth (dental caries), progressive weakness, impaired growth, increased bone fractures, muscle cramps, short, stature (adults less than 5 feet tall), skeletal deformities like asymmetrical or odd-shaped skull, bowlegs, bumps in the ribcage (rachitic rosary), breastbone pushed forward (pigeon chest), pelvic deformities, and spine deformities (spine curves abnormally, including scoliosis or kyphosis).

During the early years of childhood, genu valgum and genu varum are common concerns for parents. These problems represent normal physiologic variations in most children. However, a few children will experience pathologic lower extremity malalignment leading to cosmetic and functional deficits. Although many exist, the most frequent causes of pathologic genu varum and genu valgum are Blount's disease and renal rickets, respectively. As suggested by Hensinger 1989 [8], genu valgum is typically associated with renal osteodystrophy because the onset of chronic renal disease generally occurs while children are in the valgus phase. Metabolic conditions such as rickets affect the entire epiphyseal plate. Treatment of genu valgum and genu varum includes observation for the lesser deformities, bracing for moderate deformities and surgical correction for the excessive deformities [8]. Present case was diagnosed as bilateral genu valgum secondary to rickets and treated with femoral medial stapling by orthopaedic surgeon.

It is believed that abscesses form when pulp is infected by bacteria invading through the enamel cracks and dentinal microcleavages in the teeth. Usually both primary and permanent teeth have dentinal dysplasia. The teeth usually show taurodontism, poorly defined lamina dura and a hypoplastic alveolar ridge.

In the present case, cracks in enamel and dentin were radiographically apparent in 36 and 46 associated with an acute abscess with no carious lesions and presence of minimal wear facets. No causative factor for its necrosis could be found. Enamel cracks in the 36 and 46 may have led to microexposures of the pulp with subsequent bacterial pulpal contamination. Similar findings were observed by Cohen and Becker [9] in the maxillary right second premolar and the mandibular left canine.
